# Deriving and validating an asthma diagnosis prediction model for children and young people in primary care

**DOI:** 10.12688/wellcomeopenres.19078.2

**Published:** 2023-09-07

**Authors:** Luke Daines, Laura J Bonnett, Holly Tibble, Andy Boyd, Richard Thomas, David Price, Steve W Turner, Steff C Lewis, Aziz Sheikh, Hilary Pinnock

**Affiliations:** 1 Asthma UK Centre for Applied Research, Usher Institute, University of Edinburgh, Edinburgh, EH8 9AG, UK; 2 Department of Biostatistics, University of Liverpool, Liverpool, L69 3GL, UK; 3 Institute of Population Health Sciences, Bristol Medical School, University of Bristol, Bristol, BS8 2PS, UK; 4 Observational and Pragmatic Research Institute, Singapore, 573969, Singapore; 5 Centre of Academic Primary Care, Division of Applied Health Sciences, University of Aberdeen, Aberdeen, AB25 2ZG, UK; 6 Child Health, University of Aberdeen, Aberdeen, AB25 2ZG, UK; 7 Women and Children Division, NHS Grampian, Aberdeen, AB25 2ZG, UK; 8 Edinburgh Clinical Trials Unit, Usher Institute, University of Edinburgh, Edinburgh, EH16 4UX, UK

**Keywords:** asthma, diagnosis, primary care, children and young people, prediction model, ALSPAC, electronic health records

## Abstract

**Introduction:** Accurately diagnosing asthma can be challenging. We aimed to derive and validate a prediction model to support primary care clinicians assess the probability of an asthma diagnosis in children and young people.

**Methods:** The derivation dataset was created from the Avon Longitudinal Study of Parents and Children (ALSPAC) linked to electronic health records. Participants with at least three inhaled corticosteroid prescriptions in 12-months and a coded asthma diagnosis were designated as having asthma. Demographics, symptoms, past medical/family history, exposures, investigations, and prescriptions were considered as candidate predictors. Potential candidate predictors were included if data were available in ≥60% of participants. Multiple imputation was used to handle remaining missing data. The prediction model was derived using logistic regression. Internal validation was completed using bootstrap re-sampling. External validation was conducted using health records from the Optimum Patient Care Research Database (OPCRD).

**Results:** Predictors included in the final model were wheeze, cough, breathlessness, hay-fever, eczema, food allergy, social class, maternal asthma, childhood exposure to cigarette smoke, prescription of a short acting beta agonist and the past recording of lung function/reversibility testing. In the derivation dataset, which comprised 11,972 participants aged <25 years (49% female, 8% asthma), model performance as indicated by the C-statistic and calibration slope was 0.86, 95% confidence interval (CI) 0.85–0.87 and 1.00, 95% CI 0.95–1.05 respectively. In the external validation dataset, which included 2,670 participants aged <25 years (50% female, 10% asthma), the C-statistic was 0.85, 95% CI 0.83–0.88, and calibration slope 1.22, 95% CI 1.09–1.35.

**Conclusions:** We derived and validated a prediction model for clinicians to calculate the probability of asthma diagnosis for a child or young person up to 25 years of age presenting to primary care. Following further evaluation of clinical effectiveness, the prediction model could be implemented as a decision support software.

## Introduction

Accurately diagnosing asthma in children and young people can be challenging. Misdiagnosis is common ^1, 2^, and can lead to incorrect treatment, ongoing morbidity and the potential for disease progression. In children and young people, asthma can be difficult to diagnose for several reasons. Asthma is a heterogeneous condition with different underlying disease processes and several phenotypes ^3^. There are no definitive diagnostic tests which can accurately identify asthma in every situation ^4^. Performing tests to measure lung function and airway inflammation using spirometry (with reversibility), peak expiratory flow charting, bronchial provocation and fractional exhaled nitric oxide (FeNO) are generally recommended ^3– 6^. However, in primary care, the availability of tests can vary ^7, 8^, and in keeping with the variable nature of asthma, symptoms or lung function may have improved before testing is performed leading to false negative results ^4^. In addition, whilst largely achievable in children over seven years, performing spirometry and FeNO may be difficult for some younger children ^9^.

A clinical prediction model could help to improve the accuracy of an asthma diagnosis in primary care by determining the most valuable combination of predictors from a clinical assessment, providing a probability of asthma based on available clinical information. We previously identified seven prediction models for asthma diagnosis in primary care, including one derived for children up to 18 years old ^10^. All seven models were found to be at high risk of bias, principally due to the choice of participant selection, outcome or analysis used and were subsequently considered unreliable for informing practice ^10– 12^. Given the high risk of bias associated with existing models, and with only one prediction model available for children, we aimed to adhere closely to prediction modelling standards to derive and validate a clinical prediction model to support health professionals to assess the probability of an asthma diagnosis in children and young people presenting with symptoms suggestive of asthma in primary care.

## Methods

The study protocol was published in advance ^13^. The Transparent Reporting of a multivariable prediction model for Individual Prognosis Or Diagnosis (TRIPOD) ^14^ guided reporting (see *Extended data ^15^*).

### Derivation

***Data source and participants.*** Participant-reported data from the Avon Longitudinal Study of Parents and Children (ALSPAC) study with linked primary care electronic health records (EHR) were used to derive the model. ALSPAC is a prospective observational study that recruited pregnant women resident in and around the City of Bristol, UK with expected dates of delivery between 1st April 1991 to 31st December 1992 ^16, 17^. The offspring from the pregnancies were enrolled in the study and have been followed-up since birth. The initial number of pregnancies enrolled was 14,541. Of the initial pregnancies, there was a total of 14,676 foetuses, resulting in 14,062 live births and 13,988 children who were alive at 1 year of age. At age 18 years, study children were sent 'fair processing' materials describing ALSPAC’s intended use of their health and administrative records and were given the opportunity to object to linked data extraction from their EHR ^18^. For the derivation dataset, the inclusion criteria were participants: recruited into the initial birth cohort; alive at one year; where permissions existed for their linked EHR to be used and for whom a linkage was established to their NHS records in England and Wales. ALSPAC data are documented in a data dictionary ( http://www.bristol.ac.uk/alspac/researchers/our-data/).

***Outcome.*** Diagnostic tests were available in less than half of participants and conducted during pre-scheduled clinics rather than when clinically indicated ^3, 4^. Therefore, we defined asthma as the occurrence of at least three inhaled corticosteroid (ICS) prescriptions in one year and a ‘specific’ asthma Read code ^19^ (See Extended data ^15^ for the code list). Participants who received at least three prescriptions of an ICS (which, if used every day would typically last one month), as a single inhaler or combined with a long-acting beta agonist, on separate days within a one-year period were identified. From this group, participants who had an asthma ‘specific’ Read code (according to the validated code list from Nissen *et al.*) ^19^ occurring at any time in their patient record were selected. The event-date for those with the outcome was taken as the date at which the first of the ICS prescriptions was recorded. As participants without the outcome had no equivalent event-date, they were assigned an event-date at random. Aside from age-at-event, the outcome was developed blind to information about predictors.

***Predictors.*** Prior to modelling, potential candidate predictors were identified in ALSPAC based on our systematic review ^10^ and discussion of their value within the research team. We sought to include variables available in routine (UK) primary care. From the long list ( Table 1), predictors missing in more than 40% of participants were excluded. The 40% threshold was based on an earlier study which demonstrated that multiple imputation produced valid estimates for datasets with up to 40% missing data ^20^ and further corroborated in ALSPAC ^21^. Where predictors were correlated, the predictor that best captured the information sought, as judged by the research team, was retained. We based our decision firstly on clinical relevance, and if variables were considered equally relevant, chose the predictor with least missingness. The following candidate predictors were selected for inclusion in the modelling: sex, social class (based on the Registrar General’s Social Classes ^22^), wheeze, cough, breathlessness, hay-fever, eczema, allergy to food/drink, exposure to cigarette smoking in childhood, exposure to household mould, maternal asthma, evidence of lung function/reversibility testing having been conducted (rather than the actual result), and short acting beta agonist (SABA) prescription. A predictor was considered present if recorded (in ALSPAC or linked EHR) prior to the event date. Predictor definitions are in Table 1. Selection of predictors was made without knowing how predictors related to the outcome. Univariable modelling before multiple imputation allowed exploration of how candidate predictors related to the outcome in ALSPAC but was not used to select predictors before modelling.

**Table 1.** Candidate predictors considered for inclusion in the prediction modelling.

Variable	Variable definition	Age of child when data was collected ^#^	Selected for modelling?	Rationale for inclusion	Rationale for exclusion
***Demographics***					
Sex	Participant sex	Core dataset	Yes	Incidence of asthma varies by sex	-
Social Class	Taken from the child’s parent(s) social class measured by Registrar General Social Classes.	4 months	Yes	Potentially related to asthma	-
Ethnicity	Ethnicity of parents was available but not the child.	-	No	-	Participant ethnicity was unavailable.
Age	Age of participant at the event- date.	-	No	-	Participants without the outcome were allocated an age at event randomly.
Anthropometric indices	Height and weight measurements.	-	No	-	Height and weight were unavailable at the event date.
***Symptoms***					
Wheeze	Has your child had any periods when there was wheezing with whistling on his/her chest when he/she breathed? (Yes/No)	6, 18, 30, 42, 57, 69, 81, 91, 103, 128, 140, 157, 166, 198 and 216 months	Yes	Hallmark symptom of asthma	-
Cough at night	Has your child had a dry cough at night apart from cough associated with a cold or chest infection? (Yes/No)	81, 103, 128, 216 and 264 months	No	-	>40% of participants missing data
Cough	Has your child had a cough for which you saw a doctor about? (Yes/No)	24, 42, 69, 81, 91, 103, 128, 157 and 166 months	Yes	Hallmark symptom of asthma	-
Breathlessness	Has your child had breathlessness for which you saw a doctor about? (Yes/No)	6, 18, 30, 42, 57, 69, 81, 91, 103, 128, 157 and 166 months	Yes	Hallmark symptom of asthma	-
***Existing medical conditions***					
Hay fever	Has your child ever had hay fever? (Yes/No)	81, 91, 103, 128, 157, 166, 198 months	Yes	Atopic condition related to asthma	-
Eczema	Has your child ever had eczema? (Yes/No)	81, 91, 103, 128, 157, 166, 198 and 264 months	Yes	Atopic condition related to asthma	-
Allergy to food or drink	Are there any foods or drinks that your child is allergic to? (Yes/No)	30, 42, 54, 65, 81, 103 and 157 months	Yes	Allergy can be related to asthma	-
Allergy to a substance other than food or drink	Apart from food and drink are there any other things to which he/she is allergic? (Yes/No)	54, 65, 81, 103 and 157 months	No	-	More missing data and broader definition than allergy to food/drink.
***Exposures***					
Indoor exposure to cigarette smoke in childhood	Any regular time spent in a room or enclosed place where people are smoking? (Yes/No)	6, 24, 38, 54, 65, 77 and 103 months	Yes	Potential influencing factor for lung health / respiratory symptoms	-
Maternal smoking during pregnancy	How many cigarettes per day are you [Mother] smoking at the moment (8 or 32 weeks gestation)?	2 and 8 months gestation	No	-	No additional benefit and less acceptable to ask about than cigarette smoke exposure in childhood. 91% of children exposed to smoke during pregnancy were also exposed to smoke during childhood.
Exposure to household mould	Is mould a problem in your home? (Yes/No)	8, 21, 33, 61 and 85 months	Yes	Potentially related to asthma	-
***Family history***					
Maternal asthma	Have you [Mother] ever had asthma (Yes/No)	3 months gestation	Yes	More strongly associated with child asthma than paternal asthma	-
Paternal asthma	Have you [Father] ever had asthma (Yes/No)	3 months gestation	No	-	More missing data and less strongly associated than maternal asthma
Maternal atopy	Have you [Mother] ever had one of the following problems: Eczema, Hay fever, Allergy? (Yes/No)	3 months gestation	No	-	Combines hay fever/eczema/ allergy - less clear than maternal asthma
Paternal atopy	Have you [Father] ever had one of the following problems: Eczema, Hay fever, Allergy? (Yes/No)	3 months gestation	No	-	High proportion of missing data and not clearly related to child asthma
***Investigations***					
Peak Expiratory Flow	Peak expiratory flow measurement	61 months	No	-	>40% of participants missing data
Spirometry	Spirometry measurements completed on a subset of children at ages ~8.5 years and ~15.5 years	102 and 186 months	No	-	>40% of participants missing data
Bronchodilator reversibility	Post-bronchodilator reversibility available in a subset of participants at age ~15.5 years	186 months	No		>40% of participants missing data
Bronchial provocation	Bronchial provocation conducted on a subset of children at ~8.5 years	102 months	No		>40% of participants missing data
Evidence of reversibility or lung function testing †	Presence of a relevant clinical code in the participants linked electronic health records *	-	Yes	Provides evidence that prior testing or reversibility had been completed	-
FeNO	FeNO measurements completed on a subset of children when aged ~15.5 years old.	186 months	No	-	>40% of participants missing data
Skin prick testing	Skin prick test conducted on a subset of children at ~7.5 years of age	90 months	No	-	>40% of participants missing data
IgE	Serum IgE was completed on a subset of children aged ~7.5 years old.	90 months	No	-	>40% of participants missing data
Blood eosinophils †	Presence of a relevant clinical code in the participants linked electronic health records	-	No	-	No suitable data
***Medication prescribing***					
Short Acting Beta Agonist †	Presence of a relevant clinical code in the participants linked electronic health records	-	Yes	Indicates a treatment for respiratory symptoms has been provided.	-
Inhaled corticosteroid †	Presence of a relevant clinical code in the participants linked electronic health records	-	No		Used in the outcome measure

^#^ Age of child when data collected (i.e. questionnaire completed) was for variables constructed from ALSPAC data only. *Code lists in the Extended data ^15^ † Variable created from EHR. All other variables were created from ALSPAC data.

***Sample size.*** Sample size was determined by the number of eligible participants from ALSPAC. With 14 candidate predictors (19 parameter levels) to be included in the modelling, the events (number of individuals with the outcome) per variable (52.3) far exceeded recommendations for sample sizes, and we therefore chose not to use more formal sample size calculations ^23^.

***Missing data.*** We chose against a complete case analysis as 5,912 (49%) participants had missing data and this would have resulted in the sample size reduced by half. Missing values from ALSPAC (not including linked EHR) could have been introduced for several reasons but in most instances, it would be anticipated that a questionnaire was not completed by a participant. Consequently, we considered missing values within variables were missing at random, and under this assumption used multiple imputation by chained equations to create 20 datasets each with 250 iterations using the 14 candidate predictors and the outcome. 20 datasets were created, rather than the default of five, to minimise loss in statistical power ^24, 25^.

***Model type.*** A logistic regression model was fitted to each imputed dataset ^14, 26^. Backward step-wise selection based on Akaike’s Information Criterion (AIC) was used to select predictors ^27^. Candidate predictors selected in ≥10 of the imputed datasets were selected for use in the final model.

***Model performance.*** Apparent model performance was calculated in each imputed dataset. Discrimination, the ability to distinguish individuals with/without the outcome, was reported using the C-statistic. Calibration, which measures agreement between model predictions and observed outcomes, was assessed visually by calibration plot (evaluated in the first imputed dataset) and by the calibration slope, and ratio of expected and observed number of events (E/O) calculated using the median from the 20 imputed datasets ^28^.

### Internal validation

Bootstrapping techniques were used to internally validate the model ^28^. The modelling process, including variable selection, was repeated in 500 samples drawn with replacements from the original sample. The bootstrap performance of the model in each bootstrap sample was assessed using the C-statistic, calibration intercept and calibration slope. The performance of the bootstrap model in the original sample (test performance) was determined and the optimism, taken as the difference between the bootstrap and test performance, was calculated ^28^. Estimates of optimism from each bootstrap sample were averaged and subtracted from the apparent performance to generate an optimism-corrected estimate of performance. The optimism-adjusted calibration slope was used as the shrinkage factor to adjust the regression coefficients of the developed model for optimism.

### External validation

***Data source and participants.*** Optimum Patient Care Research Database (OPCRD), a longitudinal EHR database, holds anonymised routinely collected, primary care records for 10.1 million patients, extracted from >700 UK-based GP practices ^29^. EHRs within OPCRD provided coded patient data available from 01 January 1965 to 31 March 2020 (last extraction date). To create a dataset of participants comparable to ALSPAC, we included individuals born during or after 1990 (as for ALSPAC), with EHR data available from birth to ≥24 years of age. The number of participants meeting the criteria determined the sample size.

***Outcome and predictors.*** Outcome, event-date, SABA prescription and lung function/reversibility testing were identified using methods as for the derivation dataset. In contrast, other predictors were identified by the presence of a relevant Read code (as defined in bespoke code lists) in participants’ EHRs. Absence of a Read code for a particular predictor was taken as absence of the condition/symptom. Following this approach meant there were no missing data in the OPCRD dataset.

***Model refitting.*** Candidate predictors social class (based on the Registrar General’s Social Classes), maternal asthma and mould exposure were unavailable in EHRs. Therefore, to validate the model in OPCRD, a pragmatic approach was used: 1) Re-fit the model in the 20 imputed derivation datasets with the unattainable variables excluded; 2) Complete the internal validation of the re-fitted model and correct for optimism by repeating the bootstrapping methods.

***Calculating predictions.*** Using the linear predictor of the re-fitted model, predicted probabilities for each participant in the OPCRD dataset were calculated. Risk groups and model updating were not completed.

### Statistical analysis

Variables in ALSPAC were prepared using SPSS (v26). The OPCRD dataset was created using Microsoft SQL Server Management Studio (v18.4). All other analyses were conducted in R (v3.5.3).

## Results

### Derivation

***Participants.*** In the derivation dataset, 11,972 participants were included, of whom 5,851 (49%) were female and 970 (9%) were in the two lowest social classes ( Table 2). A total of 994 (8%) participants had asthma according to our outcome definition. Of those with asthma, there were more males than females (54% vs 46%), and 555 (56%) had a diagnosis before 10 years old. There was little difference in social class, exposure to cigarette smoke or mould between those with and without asthma. However, a higher proportion of those with asthma had hay-fever (21% vs 8%), eczema (22% vs 11%), allergy to food and drink (22% vs 13%) and maternal asthma (17% vs 10%). Before the event date, wheeze (73% v 39%), breathlessness (47% vs 14%) and cough (59% vs 37%) were proportionally higher in those with, compared to those without asthma. A higher proportion of participants with asthma had a SABA (45% vs 7%) or evidence of lung function/reversibility testing (51% vs 7%) before the event-date.

**Table 2.** Characteristics of participants in the derivation and external validation datasets.

	Levels	Derivation dataset			External validation dataset		
		No Asthma (%)	Asthma (%)	Total	No Asthma (%)	Asthma (%)	Total
Total N (%)		10978 (92)	994 (8)	11972	2399 (90)	271 (10)	2670
Age at event-date (years)	0 – 4	2529 (23)	229 (23)	2758	693 (29)	79 (29)	772
	5 – 9	3600 (33)	326 (33)	3926	995 (41)	115 (42)	1110
	10 – 14	2640 (24)	239 (24)	2879	422 (18)	46 (17)	468
	15 – 19	1435 (13)	130 (13)	1565	235 (10)	25 (9)	260
	20 – 24	774 (7)	70 (7)	844	54 (2)	6 (2)	60
Sex	Female	5389 (49)	462 (46)	5851	1198 (50)	136 (50)	1334
	Male	5589 (51)	532 (54)	6121	1201 (50)	135 (50)	1336
Social Class *	I – least deprived	912 (8)	56 (6)	968	-	-	-
	II	3670 (33)	311 (31)	3981	-	-	-
	IIIa	2982 (27)	268 (27)	3250	-	-	-
	IIIb	1268 (12)	152 (15)	1420	-	-	-
	IV	720 (7)	64 (6)	784	-	-	-
	V – most deprived	165 (2)	21 (2)	186	-	-	-
	*Missing*	*1261 (11)*	*122 (12)*	1383	*-*	*-*	*-*
Wheeze	No	5717 (52)	179 (18)	5896	2307 (96)	247 (91)	2554
	Yes	4293 (39)	724 (73)	5017	92 (4)	24 (9)	116
	*Missing*	*968 (9)*	*91 (9)*	1059	-	-	-
Cough	No	4631 (42)	212 (21)	4843	1941 (81)	173 (64)	2114
	Yes	4024 (37)	587 (59)	4611	458 (19)	98 (36)	556
	*Missing*	*2323 (21)*	*195 (20)*	2518	-	-	-
Breathlessness	No	8417 (77)	429 (43)	8846	2381 (99)	262 (97)	2643
	Yes	1574 (14)	470 (47)	2044	18 (1)	9 (3)	27
	*Missing*	*987 (9)*	*95 (10)*	1082	-	-	-
Hay-fever	No	5806 (53)	347 (35)	6153	2296 (96)	245 (90)	2541
	Yes	856 (8)	204 (21)	1060	103 (4)	26 (10)	129
	*Missing*	*4316 (39)*	*443 (45)*	4759	-	-	-
Eczema	No	5467 (50)	345 (35)	5812	2001 (83)	203 (75)	2204
	Yes	1186 (11)	216 (22)	1402	398 (17)	68 (25)	466
	*Missing*	*4325 (39)*	*433 (44)*	4758	-	-	-
Allergy to food or drink	No	7218 (66)	587 (59)	7805	2393 (100)	268 (99)	2661
	Yes	1466 (13)	217 (22)	1683	6 (0)	3 (1)	9
	*Missing*	*2294 (21)*	*190 (19)*	2484	-	-	-
Maternal asthma	No	8803 (80)	728 (73)	9531	-	-	-
	Yes	1091 (10)	173 (17)	1264	-	-	-
	*Missing*	*1084 (10)*	93 (9)	1177	-	-	-
Indoor exposure to cigarette smoke in childhood	No	4368 (40)	361 (36)	4729	2397 (100)	271 (100)	2668
	Yes	5471 (50)	532 (54)	6003	2 (0)	0 (0)	2
	*Missing*	*1139 (10)*	*101 (10)*	1240	-	-	-
Exposure to mould	No	8899 (81)	796 (80)	9695	-	-	-
	Yes	733 (7)	85 (8)	818	-	-	-
	*Missing*	*1346 (12)*	113 (11)	1459	-	-	-
Evidence of lung function or reversibility testing	No	10258 (93)	491 (49)	10749	2309 (96)	191 (70)	2500
	Yes	720 (7)	503 (51)	1223	90 (4)	80 (30)	170
SABA prescription	No	10222 (93)	544 (55)	10766	2171 (90)	80 (30)	2251
	Yes	756 (7)	450 (45)	1206	228 (10)	191 (70)	419

*Social class by parental occupation: I = Professional, II = Managerial and technical, IIIa = Skilled non-manual, IIIb = Skilled manual, IV = Partly skilled, V = Unskilled. SABA = Short Acting Beta Agonist.

***Model development.*** Unadjusted associations of each candidate predictor with the outcome are in Table 3. Exposure to mould and sex were selected in <10 imputed datasets and excluded. Remaining predictors (wheeze, cough, breathlessness, hay-fever, eczema, food allergy, social class, maternal asthma, childhood exposure to cigarette smoke, SABA prescription and evidence of lung function/reversibility testing) were included in the final model. Equation 1 shows the unadjusted asthma diagnosis multivariable model fitted in the derivation dataset.

$$\begin{array}{l} \ln\left(\frac{p_{\left(asthma\right)}}{1-p_{\left(asthma\right)}}\right)=-4.28+0.26(SocialClassII)+0.29(SocialClassIIIa)\\ \;+0.55(SocialClassIIIb)+0.18(SocialClassIV)+0.60(SocialClassV)\\ \;+0.66(Wheeze)+0.43(Cough)+0.82(Breathlessness)\\ \;+0.15(Hayfever)+0.15(Eczema)+0.17(FoodAllergy)\\ \;+0.24(MaternalAsthma)-0.20(SmokeExposure)\\ \;+1.72(LungFunction/Reversibility)+1.13(SABA) \end{array}\;Equation 1$$

**Table 3.** Univariable and multivariable odds ratios for predictors in the asthma diagnosis model fitted in the derivation dataset.

Predictor	OR (univariable)	OR (multivariable)
Sex		
Female	1 (ref)	-
Male	1.11 (0.98, 1.27)	-
Social Class *		
I – least deprived	1 (ref)	1 (ref)
II	1.38 (1.03, 1.85)	1.30 (0.94, 1.80)
IIIa	1.46 (1.09, 1.97)	1.33 (0.96, 1.85)
IIIb	1.95 (1.42, 2.68)	1.73 (1.21, 2.47)
IV	1.45 (1.00, 2.10)	1.20 (0.78, 1.84)
V – most deprived	2.07 (1.22, 3.52)	1.83 (1.00, 3.34)
Wheeze	5.39 (4.55, 6.37)	1.94 (1.57, 2.38)
Cough	3.19 (2.71, 3.75)	1.54 (1.28, 1.86)
Breathlessness	5.86 (5.09, 6.75)	2.26 (1.90, 2.69)
Hay-fever	3.99 (3.31, 4.81)	1.16 (0.93, 1.45)
Eczema	2.89 (2.41, 3.46)	1.16 (0.95, 1.42)
Allergy to food or drink	1.82 (1.54, 2.15)	1.18 (0.97, 1.43)
Maternal asthma	1.97 (1.61, 2.29)	1.27 (1.03, 1.57)
Indoor exposure to cigarette smoke in childhood	1.18 (1.02, 1.35)	0.82 (0.70, 0.97)
Exposure to mould	1.30 (1.02, 1.64)	-
Evidence of lung function/ reversibility testing	14.60 (12.62, 16.88)	5.56 (4.66, 6.64)
SABA prescription	11.19 (9.67, 12.93)	3.11 (2.60, 3.73)

Sex and exposure to mould were not included in the final multivariable model. *Social class by parental occupation: I = Professional, II = Managerial and technical, IIIa = Skilled non-manual, IIIb = Skilled manual, IV = Partly skilled, V = Unskilled. A description of the how the variables were defined is in Table S2.

**Figure 1.**
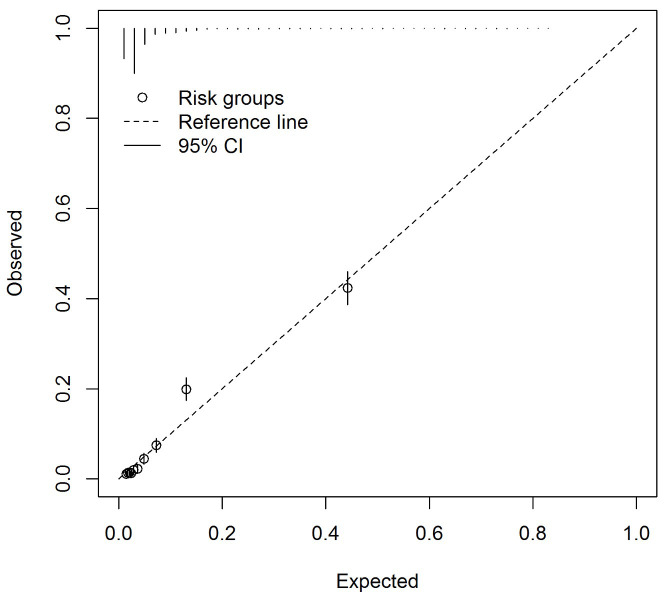
Calibration plot for the asthma diagnosis multivariable model asthma arising from imputed dataset 1 (n=11,972).

***Apparent model performance.*** The C-statistic was 0.86 (95% CI 0.85 to 0.87), indicating the model discriminated those with and without the outcome well. A calibration slope of 1.00 (95% CI 0.95 to 1.05) and E/O of 1.00 (95% CI 1.00 to 1.00) indicated a well fitted model and good calibration. The calibration plot ( Figure 1) identified mis-calibration at higher predicted probabilities though markers above and below the reference line indicated mis-calibration was not systematic.

### Internal validation

There was limited overfitting of the model in the derivation sample ( Table 4). The calibration slope adjusted-for-optimism (0.99) was used as the shrinkage factor to adjust model regression coefficients for optimism.

**Table 4.** Performance measures for the asthma diagnosis multivariable model after internal validation (derivation/internal validation dataset).

Performance measure	Original - asthma model	Optimism –asthma model	Adjusted – asthma model
**Full model**			
C statistic	0.86	0.00	0.86
Calibration slope	1	0.01	0.99
**Re-fitted model**			
C statistic	0.86	0.00	0.86
Calibration slope	1	0.01	0.99

Values displayed are the median from the 20 imputed datasets. *Full model* indicates the model built from all available candidate predictors in the derivation dataset. *Re-fitted model* indicates the model refitted without social class and maternal asthma.

### External validation

***Participants.*** A total of 2,670 participants were included in the external validation dataset, of whom 1,334 (50%) were female ( Table 2). Compared to ALSPAC, the proportion of individuals with eczema was higher (17% vs 12%), but lower for hay-fever (5% vs 9%). No relevant clinical codes were found in the external validation dataset for allergy to food or drink (0% vs 14%) or exposure to cigarette smoke during childhood (0% vs 50%). Two hundred and seventy-one (10%) participants had asthma, of whom an equal proportion were males and females (50% vs 50%). Of those with asthma, 194 (71%) had a diagnosis before 10 years of age, in contrast to 56% in ALSPAC. Cough (36% vs 19%), wheeze (9% v 4%) and breathlessness (3% vs 1%), were proportionally higher in those with asthma. A higher proportion of participants with asthma had been prescribed a SABA (70% vs 10%) or had evidence of lung function or reversibility testing (30% vs 10%) before the event-date.

**Figure 2.**
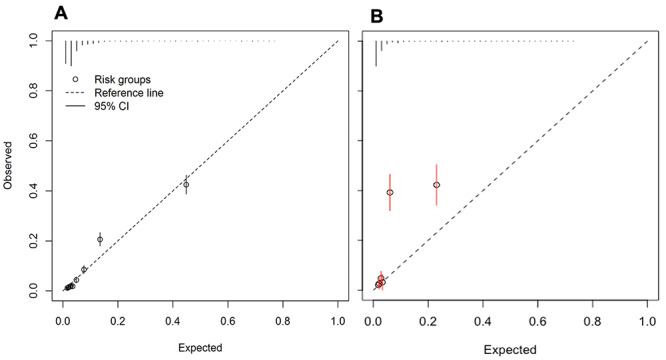
Calibration plots for the re-fitted asthma diagnosis model arising from: **A**) imputed dataset 1 (n=11,972) **B**) external validation dataset (n= 2,670).

***Model refitting.*** The re-fitted model included the same predictors as the full model, except for social class and maternal asthma. Performance of the re-fitted model was similar to the full model ( Figure 2 and Table 4), though the full model had a lower AIC than the re-fitted model (5085.93 vs 5094.33). The re-fitted model was adjusted for optimism (as shown in Equation 2) and the linear predictor used to calculate predicted probabilities of participants in the external validation dataset.

$$\begin{array}{l} \ln\left(\frac{p_{\left(asthma\right)}}{1-p_{\left(asthma\right)}}\right)=-3.97+0.66(Wheeze)+0.44(Cough)+0.83(Breathlessness)\\ \;+0.14(Hayfever)+0.14(Eczema)+0.17(FoodAllergy)\\ \;-0.14(SmokeExposure)+1.71(LungFunction/Reversibility)\\ \;+1.14(SABA) \end{array}\;Equation 2$$

***Model performance.*** Discrimination of the re-fitted model was similar to that observed in the derivation dataset with the C-statistic 0.85 (95% CI 0.83 to 0.88, Table 5). The calibration slope 1.22 (95% CI 1.09 to 1.35) indicated the model was underfitted in OPCRD, meaning that with the information available, model predictions did not adequately predict those at high probability (also visualised in the calibration plot, Figure 2).

**Table 5.** Performance of the re-fitted asthma diagnosis model in the derivation dataset and external dataset.

Performance measure	Derivation dataset (95% CI)	External validation dataset (95% CI)
C-statistic	0.86 (0.85 to 0.87)	0.85 (0.83 to 0.88)
E/O	1.00 (1.00 to 1.00)	0.44 (0.42 to 0.46)
Calibration slope	1.00 (0.95 to 1.05)	1.22 (1.09 to 1.35)

E/O = ratio of expected and observed number of events

## Discussion

Using data from a longitudinal cohort, we derived and validated a model to support primary care clinicians assess the probability of asthma diagnosis for children and young people. In ALSPAC, model performance was good, though the model produced less reliable predictions for those at higher probability of asthma. In OPCRD, model discrimination was similar to ALSPAC, yet the model was worse at predicting those at higher probability of asthma.

### Strengths and limitations

In contrast to the majority of previous prediction models for asthma diagnosis which were found to be at high risk of bias ^10^, our study sought to minimise the risk of bias (as laid out by PROBAST ^12^) by establishing a clear rationale for candidate selection, handling missing data, reporting model performance and conducting internal and external validation ^10, 13, 28^. The sample size, quality of recording and ability to link to EHR were strengths for using ALSPAC. Completing an external validation in OPCRD also had advantages, including the opportunity to use the same outcome measure.

However, using data from an existing study such as ALSPAC, rather than a study designed specifically for the derivation of a diagnostic model introduced limitations ^11, 12^. Though ALSPAC has a range of variables, some desired features (e.g. chest tightness) were unavailable. Other variables (e.g. FeNO) were missing in too many participants to be included.

We relied on clinical coding and prescribing to identify those with an asthma diagnosis and the date of diagnosis, but the outcome measure was unvalidated and likely to reflect the diagnostic and treatment practice of UK primary care clinicians between 1990 and 2015. Consequently, the outcome measure used may have under- or over-estimated the true number of participants with asthma and contributed to a higher-than-expected number of children identified with the outcome below five years of age. The decision to include a broad range of ages was pragmatic, but the downsides of this approach include missing an opportunity to consider differences in presentation by age, and including children under five, an age group in which making a diagnosis of asthma is known to be challenging. 

The aim of the study was to derive a model for use when a child or young person presented with symptoms suggestive of asthma in primary care, yet this was not possible in the chosen dataset because the sample contained individuals with and without respiratory symptoms. Consequently, further evaluation of model performance in a sample of participants presenting with symptoms is warranted as model discrimination could be worse if used only in symptomatic individuals. In addition, the predictors relied on questionnaire data collected prior to the event date, rather than at the time of diagnosis so predictors reflect the occurrence of symptoms/conditions at any time before the event date, rather than at the time of presentation with symptoms. The inclusion of predictors capturing information at the time of presentation (e.g., frequency and variation of symptoms or triggers) should be considered in future prospective research.

We sought to externally validate the model in routinely collected data, because we hoped to learn how the model would perform in a dataset which closely represented routine primary care. However, the external validation dataset had substantial differences in the way that predictors were constructed and participant characteristics, which made it harder to directly compare model performance to the derivation dataset. In OPCRD, we made the assumption that the absence of a Read code equated to the absence of a predictor. Whilst pragmatic, this approach underestimated participant characteristics, particularly wheeze, breathlessness, allergy, and smoke exposure. Lack of data for these characteristics contributed to the inferior calibration observed in OPCRD. 

### Interpretation

Similar to existing models for asthma diagnosis in primary care, our model included predictors encompassing symptoms, past medical history, family history, but also took account of social class, exposure to cigarette smoke and past treatment. A lack of appropriate data prevented us including results of investigations, as achieved previously in three models for adults ^30– 32^.

In our study, childhood exposure to cigarette smoke was associated with a reduced probability of asthma which was unexpected given that a meta-analysis found household exposure to tobacco smoke was associated with an increased incidence of asthma in children ^33^. Passive inhalation of smoke may have an inflammatory effect on the airway which can increase the likelihood of lower respiratory tract infection ^34^ and the propensity to wheeze ^35^. Therefore, in our model, exposure to cigarette smoke may have reduced the probability of asthma because an alternative reason for symptoms was more likely. Another explanation might be reverse causation, as it is possible that parents stopped smoking when their child developed asthma like symptoms.

Two predictors, evidence of lung function/ reversibility testing (which indicated that testing had been done regardless of the result) and SABA prescription prior to the index date, were strongly associated with the outcome. The presence of these codes may have indicated a diagnosis had been made but was not identified using the outcome measure. Alternatively, these variables may reflect that a clinician had previously considered the diagnosis, but at the time of testing lung function was normal or there was not enough evidence to commit to a diagnosis, so symptomatic treatment was provided.

The only prior model for asthma diagnosis in children in primary care, used healthcare provider decision as the outcome ^36^. In our study, the outcome used the presence of asthma-specific Read codes ^19^ in combination with ICS prescribing. Not all children/young people with asthma will require treatment with regular ICS and therefore it is possible that our use of this outcome measure limits the generalisability of model predictions to primary care populations. On the other hand, most contemporary guidelines recommend ICS as the first line treatment for all but the mildest forms of asthma and there has been a trend to move away from traditional labelling of individuals with the umbrella term asthma ^37, 38^. As eosinophilic airway inflammation is one of the most common and treatable phenotypes, being able to identify ‘steroid responsive asthma’ in individuals presenting with respiratory symptoms is valuable ^39^. Therefore, a possible advantage of our prediction model is that it can guide decisions on the probability of individuals having asthma that require at least three ICS prescriptions in the following 12 months. We acknowledge that further evaluation is required before the model can be used routinely ^37, 38^.

### Implications for practice and research

The model has been designed with the intention for use by health professionals to calculate the probability of asthma diagnosis for a child or young person up to 25 years of age presenting to primary care. To facilitate use, the prediction model has been incorporated into a prototype clinical decision support system (CDSS), which provides an interface for relevant predictors to be collected, and the probability calculated and visualised. The CDSS can interact with primary care EHR meaning that relevant predictors can be auto-populated in addition to being inputted by the user. As making a diagnosis of asthma in pre-schoolers is particularly challenging ^4^, the CDSS has been designed for use in children/young people aged five to 25 years. Before the prediction model could be implemented in routine clinical practice (as a CDSS or otherwise), researchers should consider assessing model performance in sub-groups of participants, completing further external validation and assessing clinical effectiveness of the model through a clinical trial of participants presenting to primary care with undifferentiated symptoms ^40^. In addition researchers should consider opportunities for using free text from EHR which could enhance the accuracy of information available from routinely collected data and improve model calibration ^41^.

## Conclusions

Making a secure diagnosis of asthma remains challenging for clinicians, especially in primary care. With further evaluation of clinical effectiveness, our model, derived from a birth cohort and externally validated in a primary care database could support clinicians assess the probability of asthma in children and young people.

### Ethics and governance

Ethical approval was achieved from the ALSPAC Law and Ethics Committee (Reference: 2018-3730) and NHS Health Research Authority - North West - Haydock Research Ethics Committee, (Reference: 10/H1010/70). Informed consent for the use of data collected via questionnaires and clinics was obtained from participants following the recommendations of the ALSPAC Ethics and Law Committee at the time. OPCRD has been ethically approved by the NHS Health Research Authority to hold and process anonymised data as part of their service delivery (Research Ethics Committee reference: 20/EM/0148). The ethical approval achieved by OPCRD covers the use of anonymised data from OPCRD in individual projects (including this study) subject to a successful review by the Anonymised Data Ethics Protocols and Transparency (ADEPT) committee – the independent scientific advisory committee for the OPCRD. The protocol for the external validation was approved by the ADEPT committee (Reference: ADEPT0320)

## Data Availability

***ALSPAC***. ALSPAC data access is through a system of managed open access. The steps below highlight how to apply for access to the data referred to in this article and all other ALSPAC data. The datasets presented in this article are linked to ALSPAC project number B2830, please quote this project number during your application. The ALSPAC variable codes highlighted in the dataset descriptions can be used to specify required variables. Please read the ALSPAC access policy ( https://www.bristol.ac.uk/media-library/sites/alspac/documents/researchers/data-access/ALSPAC_Access_Policy.pdf) which describes the process of accessing the data and samples in detail, and outlines the costs associated with doing so. You may also find it useful to browse our fully searchable research proposals database ( https://proposals.epi.bristol.ac.uk/), which lists all research projects that have been approved since April 2011. Please submit your research proposal for consideration by the ALSPAC Executive Committee. You will receive a response within 10 working days to advise you whether your proposal has been approved. If you have any questions about accessing ALSPAC data, please email alspac-data@bristol.ac.uk. The study website also contains details of all the data that is available through a fully searchable data dictionary: http://www.bristol.ac.uk/alspac/researchers/data-access/data-dictionary/ ***OPCRD***. Access to OPCRD is through a managed system. More details are available at https://opcrd.co.uk/ Open Science Framework: Extended data for ‘Clinical prediction model for the diagnosis of asthma in children and young people in primary care’, https://osf.io/kfz3n/ ^15^ This project contains the following extended data: AsthmaSpecific_ReadcodeList.txt (Asthma-specific read codes.) LungFunctionAndReversibility_ReadCodeList.txt (Lung function/reversibility testing read codes.) Open Science Framework: TRIPOD checklist for ‘Clinical prediction model for the diagnosis of asthma in children and young people in primary care’, https://osf.io/kfz3n/ ^15^ Data are available under the terms of the Creative Commons Attribution 4.0 International license (CC-BY 4.0)

## References

[1] Aaron SD Vandemheen KL FitzGerald JM : Reevaluation of diagnosis in adults with physician-diagnosed asthma. *JAMA.* 2017;317(3):269–279. 10.1001/jama.2016.19627 28114551

[2] Looijmans-Van den Akker I van Luijn K Verheij T : Overdiagnosis of asthma in children in primary care: a retrospective analysis. *Br J Gen Pract.* 2016;66(644):e152–7. 10.3399/bjgp16X683965 26917656 PMC4758494

[3] Global Initiative for Asthma: Global Strategy for Asthma Management and Prevention. 2022; [accessed September 2022]. Reference Source

[4] Health Improvement Scotland: BTS/SIGN British Guideline for the management of asthma.SIGN 158,2019; [Accessed September 2022]. Reference Source

[5] The National Institute for Health and Care Excellence: Asthma: Diagnosis, Monitoring and Chronic Asthma Management, NICE nG80. 2017; [Accessed September 2022]. Reference Source

[6] Gaillard EA Kuehni CE Turner S : European Respiratory Society clinical practice guidelines for the diagnosis of asthma in children aged 5-16 years. *Eur Respir J.* 2021;58(5): 2004173. 10.1183/13993003.04173-2020 33863747

[7] Akindele A Daines L Cavers D : Qualitative study of practices and challenges when making a diagnosis of asthma in primary care. *NPJ Prim Care Respir Med.* 2019;29(1): 27. 10.1038/s41533-019-0140-z 31316068 PMC6637121

[8] Daines L Lewis S Schneider A : Defining high probability when making a diagnosis of asthma in primary care: mixed-methods consensus workshop. *BMJ Open.* 2020;10(4): e034559. 10.1136/bmjopen-2019-034559 32317260 PMC7204930

[9] Lo D Beardsmore C Roland D : Spirometry and FeNO testing for asthma in children in UK primary care: a prospective observational cohort study of feasibility and acceptability. *Br J Gen Pract.* 2020;70(700):e809–e816. 10.3399/bjgp20X713033 33077507 PMC7575406

[10] Daines L McLean S Buelo A : Systematic review of clinical prediction models to support the diagnosis of asthma in primary care. *NPJ Prim Care Respir Med.* 2019;29(1): 19. 10.1038/s41533-019-0132-z 31073125 PMC6509212

[11] Moons KGM de Groot JAH Bouwmeester W : Critical appraisal and data extraction for systematic reviews of prediction modelling studies: the CHARMS checklist. *PLoS Med.* 2014;11(10): e1001744. 10.1371/journal.pmed.1001744 25314315 PMC4196729

[12] Moons KGM Wolff RF Riley RD : PROBAST: a tool to assess risk of bias and applicability of prediction model studies: explanation and elaboration. *Ann Intern Med.* 2019;170(1):W1–W33. 10.7326/M18-1377 30596876

[13] Daines L Bonnett LJ Boyd A : Protocol for the derivation and validation of a clinical prediction model to support the diagnosis of asthma in children and young people in primary care [version 1; peer review: 2 approved]. *Wellcome Open Res.* 2020;5:50. 10.12688/wellcomeopenres.15751.1 32724862 PMC7364181

[14] Collins GS Reitsma JB Altman DG : Transparent Reporting of a multivariable prediction model for Individual Prognosis Or Diagnosis (TRIPOD). *Ann Intern Med.* 2015;162(10):735–6. 10.7326/L15-5093-2 25984857

[15] Daines L : Clinical prediction model for the diagnosis of asthma in children and young people in primary care. 2020. https://osf.io/kfz3n/ 10.12688/wellcomeopenres.15751.1 PMC7364181 32724862

[16] Boyd A Golding J Macleod J : Cohort Profile: The ‘Children of the 90s’; the index offspring of The Avon Longitudinal Study of Parents and Children (ALSPAC). *Int J Epidemiol.* 2013;42(1):111–27. 10.1093/ije/dys064 22507743 PMC3600618

[17] Fraser A Macdonald-Wallis C Tilling K : Cohort Profile: The Avon Longitudinal Study of Parents and Children: ALSPAC mothers cohort. *Int J Epidemiol.* 2013;42(1):97–110. 10.1093/ije/dys066 22507742 PMC3600619

[18] Northstone K Lewcock M Groom A : The Avon Longitudinal Study of Parents and Children (ALSPAC): an update on the enrolled sample of index children in 2019 [version 1; peer review: 2 approved]. *Wellcome Open Res.* 2019;4:51. 10.12688/wellcomeopenres.15132.1 31020050 PMC6464058

[19] Nissen F Morales DR Mullerova H : Validation of asthma recording in the Clinical Practice Research Datalink (CPRD). *BMJ Open.* 2017;7(8): e017474. 10.1136/bmjopen-2017-017474 28801439 PMC5724126

[20] Nevalainen J Kenward MG Virtanen SM : Missing values in longitudinal dietary data: a multiple imputation approach based on a fully conditional specification. *Stat Med.* 2009;28(29):3657–69. 10.1002/sim.3731 19757484

[21] Madley-Dowd P Hughes R Tilling K : The proportion of missing data should not be used to guide decisions on multiple imputation. *J Clin Epidemiol.* 2019;110:63–73. 10.1016/j.jclinepi.2019.02.016 30878639 PMC6547017

[22] Bartley M : Health inequality: An introduction to concepts, theories and methods. 2nd Edition. Polity, 2016. Reference Source

[23] Peduzzi P Concato J Kemper E : A simulation study of the number of events per variable in logistic regression analysis. *J Clin Epidemiol.* 1996;49(12):1373–9. 10.1016/s0895-4356(96)00236-3 8970487

[24] Graham JW Olchowski AE Gilreath TD : How many imputations are really needed? Some practical clarifications of multiple imputation theory. *Prev Sci.* 2007;8(3):206–13. 10.1007/s11121-007-0070-9 17549635

[25] White IR Carlin JB : Bias and efficiency of multiple imputation compared with complete-case analysis for missing covariate values. *Stat Med.* 2010;29(28):2920–31. 10.1002/sim.3944 20842622

[26] Midi H Sarkar SK Rana S : Collinearity diagnostics of binary logistic regression model. *Journal of Interdisciplinary Mathematics.* 2010;13(3):253–267. 10.1080/09720502.2010.10700699

[27] Akaike H : A new look at the statistical model identification. *IEEE Trans Automat Contr.* 1974;19(6):716–23. 10.1109/TAC.1974.1100705

[28] Steyerberg EW : Clinical prediction models: a practical approach to development, validation, and updating.New York, USA: Springer Science & Business Media; 2009. 10.1007/978-0-387-77244-8

[29] Optimum Patient Care Research Database.[Accessed October 2022]. Reference Source

[30] Schneider A Wagenpfeil G Jörres RA : Influence of the practice setting on diagnostic prediction rules using FENO measurement in combination with clinical signs and symptoms of asthma. *BMJ Open.* 2015;5(11): e009676. 10.1136/bmjopen-2015-009676 26603255 PMC4663408

[31] Metting EI In 't Veen JCCM Dekhuijzen PNR : Development of a diagnostic decision tree for obstructive pulmonary diseases based on real-life data. *ERJ Open Res.* 2016;2(1):00077–2015. 10.1183/23120541.00077-2015 27730177 PMC5005160

[32] Louis G Schleich F Guillaume M : Development and validation of a predictive model combining patient-reported outcome measures, spirometry and exhaled nitric oxide fraction for asthma diagnosis. *ERJ Open Res.* 2023;9(1):00451–2022. 10.1183/23120541.00451-2022 36755965 PMC9900444

[33] Burke H Leonardi-Bee J Hashim A : Prenatal and passive smoke exposure and incidence of asthma and wheeze: systematic review and meta-analysis. *Pediatrics.* 2012;129(4):735–44. 10.1542/peds.2011-2196 22430451

[34] Li JS Peat JK Xuan W : Meta-analysis on the association between environmental tobacco smoke (ETS) exposure and the prevalence of lower respiratory tract infection in early childhood. *Pediatr Pulmonol.* 1999;27(1):5–13. 10023785 10.1002/(sici)1099-0496(199901)27:1<5::aid-ppul3>3.0.co;2-5

[35] Lux AL Henderson AJ Pocock SJ : Wheeze associated with prenatal tobacco smoke exposure: a prospective, longitudinal study. ALSPAC Study Team. *Arch Dis Child.* 2000;83(4):307–12. 10.1136/adc.83.4.307 10999864 PMC1718491

[36] Hall CB Wakefield D Rowe TM : Diagnosing pediatric asthma: validating the Easy Breathing Survey. *J Pediatr.* 2001;139(2):267–72. 10.1067/mpd.2001.116697 11487755

[37] Pavord ID Beasley R Agusti A : After asthma: redefining airways diseases. *Lancet.* 2018;391(10118):350–400. 10.1016/S0140-6736(17)30879-6 28911920

[38] Agusti A Bel E Thomas M : Treatable traits: toward precision medicine of chronic airway diseases. *Eur Respir J.* 2016;47(2):410–9. 10.1183/13993003.01359-2015 26828055

[39] Drake SM Simpson A Fowler SJ : Asthma diagnosis: the changing face of guidelines. *Pulm Ther.* 2019;5(2):103–115. 10.1007/s41030-019-0093-y 32026404 PMC6967246

[40] Wallace E Smith SM Perera-Salazar R : Framework for the impact analysis and implementation of Clinical Prediction Rules (CPRs). *BMC Med Inform Decis Mak.* 2011;11(1): 62. 10.1186/1472-6947-11-62 21999201 PMC3216240

[41] Nicholson A Tate AR Koeling R : What does validation of cases in electronic record databases mean? The potential contribution of free text. *Pharmacoepidemiol Drug Saf.* 2011;20(3):321–4. 10.1002/pds.2086 21351316 PMC3083518

