# Supplementary material for: Deriving and validating an asthma diagnosis prediction model for children and young people in primary care
Source: Wellcome Open Res. 2023 Sep 7;8:195. Originally published 2023 May 3. [Version 2] doi: 10.12688/wellcomeopenres.19078.2 (PMC10622861; doi:10.12688/wellcomeopenres.19078.2)
Supplement: Peer review history

# Reviewer report: Reviewer response for version 2

**DOI:** 10.21956/wellcomeopenres.22173.r66685

**Published:** 2023-11-02

**Authors:** Claudia Kuehni, Eva Pedersen

**Affiliations:** 1 Institute of Social and Preventive Medicine, Paediatric Epidemiology, Universitat Bern, Bern, Canton of Bern, Switzerland; 2 ISPM, University of Bern, Bern, Canton of Bern, Switzerland

Thank you for the revised manuscript, in which all the points we raised are now discussed. However, only few comments were actually implemented in the prediction model that is presented.

 We of course understand that it is a lot of work to make changes to the fundamental model, but we still strongly believe that the model would be more useful for physicians if the inclusion criteria resembled more the population who would be evaluated for asthma in the first place, e.g. children with cough or wheeze – at a specific time or at any time perhaps. Alternatively, the authors could run a sensitivity analysis in a restricted sample and study predictive performance of the model.  A model, which includes mainly healthy children is not really useful for the clinician, who wants to assess the asthma risk in children who visit him for asthma-like symptoms. Such a model is also likely to overperform, as it includes much heterogeneity, and of course it is always easier to distinguish healthy  children form sick children, than to predict one of competing diagnoses in children presenting with relatively  non-specific symptoms. As the datasets are large, we believe they would have allowed to run an analysis including only symptomatic children. The same relates to age-specific or age-stratified models. Given that the diagnosis and phenotypes of asthma vary greatly over the growth period, it seems unlikely that models including all people from infancy to age 25 years might be clinically really useful.

 Also, we would propose to show a risk score based on the developed prediction model ,with performance measures (e.g. sensitivity, positive predictive values) for each score cut-off which could be used by clinicians.

 Saying this, the paper is carefully presented, it describes how the model was developed (this is in stark contrast to much that has been published previously) and limitations are discussed.

Is the work clearly and accurately presented and does it cite the current literature?

Yes

If applicable, is the statistical analysis and its interpretation appropriate?

Yes

Are all the source data underlying the results available to ensure full reproducibility?

Partly

Is the study design appropriate and is the work technically sound?

Yes

Are the conclusions drawn adequately supported by the results?

Partly

Are sufficient details of methods and analysis provided to allow replication by others?

Partly

Reviewer Expertise:

Paediatrics (primary care), paediatric pulmonology, epidemiology

We confirm that we have read this submission and believe that we have an appropriate level of expertise to confirm that it is of an acceptable scientific standard.

# Reviewer report: Reviewer response for version 2

**DOI:** 10.21956/wellcomeopenres.22173.r66684

**Published:** 2023-10-02

**Authors:** Rachel E. Foong

**Affiliations:** 1 Telethon Kids Institute, Nedlands, Western Australia, Australia

No further comments to make.

Is the work clearly and accurately presented and does it cite the current literature?

Yes

If applicable, is the statistical analysis and its interpretation appropriate?

Yes

Are all the source data underlying the results available to ensure full reproducibility?

Yes

Is the study design appropriate and is the work technically sound?

Yes

Are the conclusions drawn adequately supported by the results?

Partly

Are sufficient details of methods and analysis provided to allow replication by others?

Partly

Reviewer Expertise:

Respiratory physiology, biostatistics, epidemiology

I confirm that I have read this submission and believe that I have an appropriate level of expertise to confirm that it is of an acceptable scientific standard.

# Reviewer report: Reviewer response for version 1

**DOI:** 10.21956/wellcomeopenres.21152.r60017

**Published:** 2023-06-28

**Authors:** Rachel E. Foong

**Affiliations:** 1 Telethon Kids Institute, Nedlands, Western Australia, Australia

Daines *et al.* undertook a study aiming to develop an asthma prediction model for individuals under 25 years of age. They used data from a large UK-based longitudinal birth cohort study to develop the prediction model, then used this model as a basis for asthma prediction in patients within an electronic health record database. The model was developed using known asthma predictors identified through the literature, and showed good predictive performance based on the C-statistic for logistic regression models in the original ALSPAC dataset, however, did not perform as well for predicting asthma in the EHR dataset. Based on these findings, the authors conclude that a prediction model encompassing predictors wheeze, cough, breathlessness, hay-fever, eczema, food allergy, social class, maternal asthma, childhood exposure to cigarette smoke, SABA prescription and evidence of lung function/reversibility testing could guide the management of individuals with asthma that requires at least three ICS prescriptions in the following 12 months.

 The ability to predict asthma in young children has been a goal of many research teams. This study has however taken a different approach by including a large age range including individuals up to 25 years of age. The study does take a novel approach of aiming to validate their findings in a large dataset based on a population within an EHR database. Comments are as follows:

1. While the authors present their findings as taking a 'treatable traits' approach where they conclude that their findings are applicable to individuals with asthma who require at least three ICS prescriptions in the following 12 months, the predictors identified are known within the literature to be associated with asthma and may be common to other 'treatable traits' not assessed here. Without assessing another 'treatable trait' of asthma, can the authors be certain this prediction model is not applicable to individuals with asthma without the ICS prescription for example. Can this be assessed?
2. The asthma Read code used in determining the outcome should be clearly defined. The authors provide a reference to Nissen *et al. *2017, however this study outlines a comparison of various combinations of the asthma Read code with reversibility testing and asthma medications, to confirm asthma diagnosis, with positive predictive values around 86%, suggesting the possibility of inaccuracies as well. Are the authors confident that the predictors are not included in the asthma Read code used to define the outcome?
3. The authors used the C-statistic to conclude that the model could discriminate those with asthma and those without. Previous childhood prediction models have been found to have good specificity or negative predictive value, but low sensitivity or positive predictive value. It would be helpful to be able to differentiate this as well in this model. Is there good sensitivity?
4. With regards to the rationale for excluding the predictor Allergy to a substance other than food or drink. Allergies to aeroallergens such as house dust mite and pet dander may have additional benefit? Similarly, I would argue that Maternal smoking during pregnancy does provide additional information to cigarette smoke exposure in childhood. The extent of exposure in childhood may be variable depending if the exposure was indoors, outdoors or in a vehicle, however exposure in utero can affect lung development.

Is the work clearly and accurately presented and does it cite the current literature?

Yes

If applicable, is the statistical analysis and its interpretation appropriate?

Yes

Are all the source data underlying the results available to ensure full reproducibility?

Yes

Is the study design appropriate and is the work technically sound?

Yes

Are the conclusions drawn adequately supported by the results?

Partly

Are sufficient details of methods and analysis provided to allow replication by others?

Partly

Reviewer Expertise:

Respiratory physiology, biostatistics, epidemiology

I confirm that I have read this submission and believe that I have an appropriate level of expertise to confirm that it is of an acceptable scientific standard, however I have significant reservations, as outlined above.

# Response

**Published:** 2023-09-04

**Authors:** Luke Daines

**Affiliations:** University of Edinburgh, UK

Thank you for reviewing our manuscript and your helpful comments which we have responded to point by point below.

- While the authors present their findings as taking a 'treatable traits' approach where they conclude that their findings are applicable to individuals with asthma who require at least three ICS prescriptions in the following 12 months, the predictors identified are known within the literature to be associated with asthma and may be common to other 'treatable traits' not assessed here. Without assessing another 'treatable trait' of asthma, can the authors be certain this prediction model is not applicable to individuals with asthma without the ICS prescription for example. Can this be assessed?

**Response: **Thank you for letting us elaborate on this point. We feel that the model could be applicable to those with a different treatable trait and with hindsight, feel that our use of the term ‘treatable trait’ has been unhelpful here. We weren’t trying to indicate that the outcome only identified those (for example) with sputum eosinophilia. Rather, it was a suggestion to distinguish that the outcome couldn’t be considered as being all phenotypes of asthma. We realise that our use of the term treatable trait may have raised specific implications which wasn’t our intention. Therefore, we have edited the discussion as follows: *“* *In our study, the outcome used the presence of asthma-specific Read codes in combination with ICS prescribing. Not all children/young people with asthma will require treatment with regular ICS and therefore it is possible that our use of this outcome measure limits the generalisability of model predictions to primary care populations. On the other hand, most contemporary guidelines recommend ICS as the first line treatment for all but the mildest forms of asthma and there has been a trend to move away from traditional labelling of individuals with the umbrella term asthma. As eosinophilic airway inflammation is one of the most common and treatable phenotypes, being able to identify ‘steroid responsive asthma’ in individuals presenting with respiratory symptoms is valuable. Therefore, a possible advantage of our prediction model is that it can guide decisions on the probability of individuals having asthma that require at least three ICS prescriptions in the following 12 months. We acknowledge that further evaluation is required before the model can be used routinely.* *”*

- The asthma Read code used in determining the outcome should be clearly defined. The authors provide a reference to Nissen *et al. *2017, however this study outlines a comparison of various combinations of the asthma Read code with reversibility testing and asthma medications, to confirm asthma diagnosis, with positive predictive values around 86%, suggesting the possibility of inaccuracies as well. Are the authors confident that the predictors are not included in the asthma Read code used to define the outcome?

**Response: **As per the journal requirements the code list we used for the outcome measure and previous lung function/reversibility testing is available in the extended data. Nissen et al., 2017 did indeed use different methods of identifying asthma diagnosis. Following correspondence with the authors we chose to use the asthma ‘specific’ Read codes only. The code list we used for previous lung function/reversibility testing was based on the reversibility codes specified by Nissen et al., but adds other codes relevant to the broader concept of lung function testing. The codes contained in the separate code lists are distinct. We have added a reference to the extended data in the Methods: *“Therefore, we defined asthma as the occurrence of at least three inhaled corticosteroid (ICS) prescriptions in one year and a ‘specific’ asthma Read code* *^19^* *^^(See Extended data ^15^ for the code list).”*

- The authors used the C-statistic to conclude that the model could discriminate those with asthma and those without. Previous childhood prediction models have been found to have good specificity or negative predictive value, but low sensitivity or positive predictive value. It would be helpful to be able to differentiate this as well in this model. Is there good sensitivity?

**Response: **As for our response to reviewer 1, we chose to prioritise c-statistic, calibration slope, calibration plot based on the prediction modelling training received from experts from the TRIPOD group. ^5^ Calculating other measures of performance such as sensitivity, specificity, negative and positive predictive values would have been another approach but would require a threshold / risk groups to be defined which we chose not to do.

- With regards to the rationale for excluding the predictor Allergy to a substance other than food or drink. Allergies to aeroallergens such as house dust mite and pet dander may have additional benefit? Similarly, I would argue that Maternal smoking during pregnancy does provide additional information to cigarette smoke exposure in childhood. The extent of exposure in childhood may be variable depending if the exposure was indoors, outdoors or in a vehicle, however exposure in utero can affect lung development.

**Response: **Allergies to aeroallergens: We agree with you about the relevance of allergy to aeroallergens for asthma and did include hay fever (a clinical manifestation of being affected by an aeroallergen) as a predictor variable. The decision to exclude allergy to substance other than food or drink was based on the following considerations. Firstly, we considered the question asked in the ALSPAC questionnaire (Apart from food and drink are there any other things to which he/she [the child] is allergic?) was a bit imprecise and if included in the model might be confusing for clinicians to ask about. Secondly, it had more missing data than for the allergy to food and drink variable (36% participants with missing data compared to 21%). We have updated Table 1 to provide a greater explanation for the decision. Maternal smoking: Again, we agree that Maternal smoking during pregnancy does offer slightly different information. To clarify, the exposure to cigarette smoke was based on the question, “Please indicate how often during the day the child is in a room or enclosed place where people are smoking”, which we turned into a binary variable. Our decision not to include maternal smoking during pregnancy as well as exposure to smoking in childhood was based on the following: 91% of children who were exposed to smoke during pregnancy were also exposed to smoke during childhood. 47% of children not exposed to smoking during pregnancy were exposed to cigarette smoke during childhood, making it potentially difficult to interpret the value of smoking during pregnancy in these children. Also, from a clinical perspective we felt that asking a parent about exposure during childhood would be more acceptable (and less impacted by social desirability bias) than during pregnancy. Exposure during childhood would also be easier to answer by a young person/teenager if they presented for consultation without a parent. In light of your comment, we have updated Table 1 to clarify that it was indoor smoke exposure and to provide greater explanation for the decision.

# Reviewer report: Reviewer response for version 1

**DOI:** 10.21956/wellcomeopenres.21152.r56900

**Published:** 2023-05-31

**Authors:** Claudia Kuehni, Eva Pedersen

**Affiliations:** 1 Institute of Social and Preventive Medicine, Paediatric Epidemiology, Universitat Bern, Bern, Canton of Bern, Switzerland; 2 ISPM, University of Bern, Bern, Canton of Bern, Switzerland

This paper, which I enjoyed reading, aims to derive and externally validate an asthma diagnosis prediction model for primary care, for children and young people aged 1 to 18 years. The model is derived using ALSPAC data (combined survey data with linked medical records); N=11972) and externally validated using the OPCRD (Optimum patient care research; only data from medical records, N= 267).

 I would like to commend the authors for the high-quality work presented here, derived from a thorough preparation (review of existing models, published study protocol for developing the new model, transparent description of datasets, missingness and analysis, reporting model performance, imputing missing values using MI, performing internal and external validation). The methodology is the best I have seen among published asthma prediction models. The final model includes previous occurrence of wheeze, cough and breathlessness, and previous lung function testing and SABA use as strong predictors, and social class, maternal asthma, hayfever, eczema and food allergy as weak predictors.

 Nevertheless I have some  major concerns, most of which relate to the choice of study population and setting, and some are methodological. As I am not a statistician, I think it would be good to include in the review process a statistician with high expertise in clinical prediction models.

**Major comments:**

1. **Choice of setting and study population: **The paper aims to develop a model that can be used in primary care, to help GPs make (or exclude) a diagnosis of asthma when evaluating children aged between 1 and 18 years presenting with recurrent respiratory symptoms. The outcome (=”Diagnosis”) is defined as a child who has a GP diagnosis of asthma and needs at least 3 prescriptions for ICS per year. Thus, the target population of interest are children and young people presenting repeatedly to the GP with respiratory symptoms, the outcome is physician-diagnosed asthma needing ICS treatment for several months a year. I have several questions relating to this:
  1. **Target population:** It is easy for a GP (in fact for everybody) to distinguish children with moderately severe asthma (needing continuous inhaled steroids) from totally healthy children. This is what the model does, by including all ALSPAC children from the (mostly healthy) birth cohort. The real question for the GP (and much more difficult) is to distinguish children with asthma from other children presenting repeatedly with cough, wheeze and breathlessness (i.e. for possible asthma). This is the study population that should be included, and not the entire, mostly healthy, ALSPAC group. This is reflected by Figure 1 which shows that nearly all dots are in the left bottom corner. I think this explains the high AUC. The model would probably be worse if applied only to children presenting at the GP for possible asthma.
  2. **Age groups:** For a paediatrician it is difficult to understand why the target age range is so broad, including infants, toddlers, schoolchildren and young adults. Asthma signs and symptoms vary significantly according to age. Asthma diagnosis guidelines distinguish under fives from older children. And some of the important predictors (in particularly previous lung function testing, but also previous symptoms such as wheeze or hayfever) are not available or only available for a shorter time period in younger children. Thus, I would have expected models adapted to specific age groups, or at least interaction terms with age included in the modelling process (not sure this could be done with the approach chosen,  or would rather need decision trees). Honestly, I am not sure if an all-age-model is useful.
  3. **Source of information on predictors:** I did not understand why the derivation study included not only predictors available to GPs (obtained from families when the child is ill), but also predictors obtained in ALSPAC by use of questionnaire surveys at different time-points unrelated to current morbidity or health care visits. Table 2 shows indeed that prevalence of symptoms varies hugely between datasets (e.g. breathlessness among asthmatics 47% in ALSPAC, 3% in validation study). Some data (such as social class) might not be available to GPs who don’t have  questionnaires from their patients.
2. **Choice of outcome:** the outcome is not very robust; as it does not include any objective tests, but only reflects the diagnostic and treatment habits of GPs. Thus, if – as a GP - I use the model to help me diagnose asthma, all it does is bringing me closer to the average of other GPs (i.e. the average of GPs working in the Bristol area in the early 2000s). Thus, depending on my prior skills, the model will not always improve my work, but could also make it worse (if I have above average knowledge on asthma). This could be discussed.
3. **Choice of predictor variables:** I commend the authors for having searched the literature and taking a systematic approach for selection of predictors, looking at clinical meaning and availability of data. I have a few questions here:
  1. **Previous lung function tests and previous SABA use** will only be available for older participants (lung function), and actually reflects a previous diagnosis or differential diagnosis of asthma made by the treating physician. Given that also the outcome is physicians diagnosis (expressed in words and by ICS prescription), this seems a self-fulfilling prophecy. Of course a GP will make more often a diagnosis it the time of a visit if he has already made it previously and recorded it in the records.
  2. Could the authors justify why the they included “ **allergy” to food and drink**, which  - certainly when parent-reported – are only weakly if at all related to asthma. These children can have a range of underlying problems, and IgE mediated food allergy is relatively rare in my experience.
  3. **Maternal smoking**: I would welcome arguments why information on maternal smoking was included in the final model. If I understand, it was only weakly related, and negatively (i.e. protective). Should I, as a GP, really take this into account and tend not to treat a child with ICS if the mum smokes? could the findings be explained by reverse causation (mothers stopped smoking when children became symptomatic) or by the chosen reference standard (doctors might have tended to prescribe antibiotics rather than ICS to children of smoking mums?).  At least this should be explained.
  4. It is a pity that the model does only use information on previous symptoms (any time in life – yes/no) and previous lung function tests and prescriptions of asthma inhalers, but not more detailed information on the current health status (when the child presents to the GP to get the DX/ICS prescription, and the 12 months before. This could be more informative and improve the model further. Such clinical details (which could easily be assessed by the physician would be frequency of symptoms at day- and nigh-time, trigger factors, etc). I understand this is an inherent limitation of the dataset, but it could be further discussed in particular with relating to future research.
  5. **Final number of variables**: It seems that all variables that remained significant in a backwards selection were included in the model. I wonder why not a more robust variable selection (some shrinkage procedure, such as by the LASSO method) has been chosen, or why coefficients were not rounded. To my understanding, all this would have further reduced overfitting and made the model more robust and simpler. For instance, I am not sure if variables with very small coefficients (i.e. Table 3: smoking, social class, hayfever, eczema, allergy to food or drinks) substantially improve the model fit (sufficient improvement to justify making it more complicated).
  6. **Describing model performance**: for the clinician, it would be helpful if additional measures were shown, such as sensitivity and specificity, NPV and PPV, for different scores.
4. **External validation: **
  1. The dataset used for external validation is very different from the one used for derivation, in that only information from GP records is available, while in the derivation sample they used also information form research questionnaires.  (see discrepancies in prevalence of predictors, Table 2). I think it would have been easier (and closer to the setting the model will be used in) if also the derivation dataset used only information form the health care records.
  2. I am not sure if the process described is really an external validation. (Steyerberg EW. Clinical Prediction Models : A Practical Approach to Development Validation and Updating. New York: Springer; 2009. doi:10.1007/978-0-387-77244-8). Rather, it seems the model was re-fitted in the new dataset.
  3. In my (statistically limited) understanding it would have been more logical to use in the derivation cohort only those predictors which are available in both cohorts, fit the model only with these, and see how the original model performs in the validation cohort .

**Minor:**

1. For the reader, it would be good to shortly describe/explain  the asthma Read code in the paper
2. Could you tell the reader how many doses are contained in an average UK ICS prescription; i.e. for how many months of twice-daily treatment will the three prescriptions last?
3. Table 4: please specify if the data for this table come from the derivation or validation cohort.
4. Please describe better at which time-points the predictor data were selected, and (in ALSPAC), how much information was provided by questionnaires and by health care data.

Saying all that, the paper has been well done and transparently describe and very much deserves publication after some revision.  

 PS. I base my comments mainly on the suggestions by Steyerberg, citation below (or what I understood from it)

Is the work clearly and accurately presented and does it cite the current literature?

Yes

If applicable, is the statistical analysis and its interpretation appropriate?

Yes

Are all the source data underlying the results available to ensure full reproducibility?

Partly

Is the study design appropriate and is the work technically sound?

Yes

Are the conclusions drawn adequately supported by the results?

Partly

Are sufficient details of methods and analysis provided to allow replication by others?

Partly

Reviewer Expertise:

Paediatrics (primary care), paediatric pulmonology, epidemiology

We confirm that we have read this submission and believe that we have an appropriate level of expertise to state that we do not consider it to be of an acceptable scientific standard, for reasons outlined above.

## References

[1] : Clinical Prediction Models. 10.1007/978-3-030-16399-0 10.1007/978-3-030-16399-0

# Response

**Published:** 2023-09-04

**Authors:** Luke Daines

**Affiliations:** University of Edinburgh, UK

Thank you for reviewing our manuscript and your helpful comments which we have responded to point by point below.

**Major comments:**

**1. Choice of setting and study population: **The paper aims to develop a model that can be used in primary care, to help GPs make (or exclude) a diagnosis of asthma when evaluating children aged between 1 and 18 years presenting with recurrent respiratory symptoms. The outcome (=”Diagnosis”) is defined as a child who has a GP diagnosis of asthma and needs at least 3 prescriptions for ICS per year. Thus, the target population of interest are children and young people presenting repeatedly to the GP with respiratory symptoms, the outcome is physician-diagnosed asthma needing ICS treatment for several months a year. I have several questions relating to this:

- **Target population:** It is easy for a GP (in fact for everybody) to distinguish children with moderately severe asthma (needing continuous inhaled steroids) from totally healthy children. This is what the model does, by including all ALSPAC children from the (mostly healthy) birth cohort. The real question for the GP (and much more difficult) is to distinguish children with asthma from other children presenting repeatedly with cough, wheeze and breathlessness (i.e. for possible asthma). This is the study population that should be included, and not the entire, mostly healthy, ALSPAC group. This is reflected by Figure 1 which shows that nearly all dots are in the left bottom corner. I think this explains the high AUC. The model would probably be worse if applied only to children presenting at the GP for possible asthma.

**Response: **We agree with your points and feel that this is a notable limitation to the study. We have added the following text to the discussion: *“* *The aim of the study was to derive a model for use when a child or young person presented with symptoms suggestive of asthma in primary care, yet this was not possible in the chosen dataset because the sample contained individuals with and without respiratory symptoms. Consequently, further evaluation of model performance in a sample of participants presenting with symptoms is warranted as model discrimination could be worse if used only in symptomatic individuals* *.”*

- **Age groups:** For a paediatrician it is difficult to understand why the target age range is so broad, including infants, toddlers, schoolchildren and young adults. Asthma signs and symptoms vary significantly according to age. Asthma diagnosis guidelines distinguish under fives from older children. And some of the important predictors (in particularly previous lung function testing, but also previous symptoms such as wheeze or hayfever) are not available or only available for a shorter time period in younger children. Thus, I would have expected models adapted to specific age groups, or at least interaction terms with age included in the modelling process (not sure this could be done with the approach chosen,  or would rather need decision trees). Honestly, I am not sure if an all-age-model is useful.

**Response: **Using the ALSPAC dataset meant that data were available for study participants from birth to 25 years of age. In primary care, individuals present at any age, and we decided to make use of all the data available, rather than restrict by age. We felt this would appeal to primary care clinicians, in that the prediction model could be used for any child or young person (up to 25 years). However, we agree that the predictors selected, and the strength of association between predictors and outcome may have been different had we derived separate models by age. It was not possible to include age as a predictor because it was used in determining an event date for those without asthma. We agree that diagnosing asthma in children under five years is challenging. We have changed the limitations section as follows: *“* *We relied on clinical coding and prescribing to identify those with an asthma diagnosis and the date of diagnosis, but the outcome measure was unvalidated and likely to reflect the diagnostic and treatment practice of UK primary care clinicians between 1990 and 2015. Consequently, the outcome measure used may have under- or over-estimated the true number of participants with asthma and contributed to a higher-than-expected number of children identified with the outcome below five years of age. The decision to include a broad range of ages was pragmatic, but the downsides of this approach include missing an opportunity to consider differences in presentation by age, and including children under five, an age group in which making a diagnosis of asthma is known to be challenging* *.”* 

- **Source of information on predictors:** I did not understand why the derivation study included not only predictors available to GPs (obtained from families when the child is ill), but also predictors obtained in ALSPAC by use of questionnaire surveys at different time-points unrelated to current morbidity or health care visits. Table 2 shows indeed that prevalence of symptoms varies hugely between datasets (e.g. breathlessness among asthmatics 47% in ALSPAC, 3% in validation study). Some data (such as social class) might not be available to GPs who don’t have  questionnaires from their patients.

**Response:** Predictors captured ever having a symptom rather than at the time of presentation. Our vision was for the prediction model to be operationalised via a Clinical Decision Support System (CDSS) which would search for codes in the patient electronic health records and auto-populate the prediction model. Any features not picked up from the health records could be asked about by the clinician during the appointment. There was a large disparity in symptoms between ALSPAC and the validation study which reflects both the enriched data (questionnaires of parents and young people) in ALSPAC and, we think, symptoms being poorly coded in routinely collected data. We have added the following text in the limitations section of the discussion: *“* *In addition, the predictors relied on questionnaire data collected prior to the event date, rather than at the time of diagnosis so predictors reflect the occurrence of symptoms/conditions at any time before the event date, rather than at the time of presentation with symptoms. The inclusion of predictors capturing information at the time of presentation (e.g., frequency and variation of symptoms or triggers) should be considered in future prospective research.* *”*

**2. Choice of outcome:** the outcome is not very robust; as it does not include any objective tests, but only reflects the diagnostic and treatment habits of GPs. Thus, if – as a GP - I use the model to help me diagnose asthma, all it does is bringing me closer to the average of other GPs (i.e. the average of GPs working in the Bristol area in the early 2000s). Thus, depending on my prior skills, the model will not always improve my work, but could also make it worse (if I have above average knowledge on asthma). This could be discussed.

**Response:** In the absence of an established reference standard for asthma, and with diagnostic tests available in less than a half of participants in the sample, our choice of outcome measure was pragmatic but has limitations as it reflects GP diagnosis which is known to over- (and probably under-) diagnose asthma. Including prescription of three ICS in a year is likely to have reduced the chance of children with transient symptoms being labelled as having asthma. Using a prediction model could result in regression to the mean in terms of clinician decision making. Interestingly in our recent qualitative work (unpublished), health professionals considered that a CDSS for asthma diagnosis would more likely benefit (and be used by) less experienced clinicians or trainees. Experienced clinicians did not feel they would gain from using a decision support, which reflects your point. In light of your comments, we have added the following text to the limitations section: *“* *We relied on clinical coding and prescribing to identify those with an asthma diagnosis and the date of diagnosis, but the outcome measure was unvalidated and likely to reflect the diagnostic and treatment practice of UK primary care clinicians between 1990 and 2015. Consequently, the outcome measure used may have under- or over-estimated the true number of participants with asthma and contributed to a higher-than-expected number of children identified with the outcome below five years of age.* *”*

**3. Choice of predictor variables:** I commend the authors for having searched the literature and taking a systematic approach for selection of predictors, looking at clinical meaning and availability of data. I have a few questions here:

- **Previous lung function tests and previous SABA use** will only be available for older participants (lung function), and actually reflects a previous diagnosis or differential diagnosis of asthma made by the treating physician. Given that also the outcome is physicians diagnosis (expressed in words and by ICS prescription), this seems a self-fulfilling prophecy. Of course a GP will make more often a diagnosis it the time of a visit if he has already made it previously and recorded it in the records.

**Response: **Firstly, to clarify, the list of Read codes used for the outcome measure, previous lung function/reversibility testing and SABA use were distinct, so there was no overlap between the three variables (see the data repository for code lists: https://osf.io/kfz3n/). Secondly, the Read codes for lung function/reversibility testing included codes which indicated testing had been done, regardless of the result (i.e. even if a negative test was recorded). In relation to previous lung function/reversibility testing, as you say, it is possible that the diagnosis was made following the result of the lung function testing, which would be a natural sequence of events. Yet, it may also reflect that a clinician had considered the diagnosis before and considered or completed testing, but the result of the test did not lead to a diagnosis. We were interested in including this predictor because in primary care, it is common for lung function and reversibility testing to be falsely negative. For example, the British Thoracic Society/Scottish Intercollegiate Guideline Network asthma guideline reported that for spirometry, the negative predictive value for asthma ranged between 18 and 54% indicating that more than half of individuals with normal spirometry will in fact have asthma.[i] Therefore, the significance of the predictor is that a clinician had previously considered the diagnosis – a common occurrence in a long-term variable condition. For the SABA variable, clinicians may not wish to commit to a diagnosis of asthma at the first presentation of a child or young person with wheeze or breathlessness, and instead choose to treat symptomatically and review at a later date. Prescribing a SABA would be first choice for the symptomatic relief of wheeze again reflecting that the diagnosis of asthma had been considered. Overall, we think these variables represent that someone else in the GP practice had thought about a diagnosis of asthma before, but the lung function test was normal, or that they were provided with a symptomatic treatment. We have added the following text to the discussion: *Two predictors, evidence of lung function/ reversibility testing (which indicated that testing had been done regardless of the result) and SABA prescription prior to the index date, were strongly associated with the outcome. The presence of these codes may have indicated a diagnosis had been made but was not identified using the outcome measure. Alternatively, these variables may reflect that a clinician had previously considered the diagnosis, but at the time of testing lung function was normal or there was not enough evidence to commit to a diagnosis, so symptomatic treatment was provided.*

- Could the authors justify why the they included “ **allergy” to food and drink**, which  - certainly when parent-reported – are only weakly if at all related to asthma. These children can have a range of underlying problems, and IgE mediated food allergy is relatively rare in my experience.

**Response: **Our systematic review identified history of allergy or atopy as a commonly occurring predictor in prediction models for asthma diagnosis, so we were keen to include allergy in the candidate predictors.[ii] In ALSPAC, allergy was asked about in two ways, “Are there any foods or drinks that your child is allergic to?” and  “Apart from food and drink are there any other things to which he/she is allergic?” Given the inter-relatedness of these variables, we chose to include one “allergy” variable only. We decided to exclude ‘allergy to substance other than food or drink’ because firstly, it had more missing data than the ‘allergy to food and drink’ variable (36% participants with missing data compared to 21%). Secondly, we considered the question asked in the ALSPAC questionnaire was less clear than the ‘allergy to food and drink’ variable and if included in the model might be confusing for clinicians to ask about and patients/parents to respond to. Thirdly, hay fever was also included as a candidate predictor and is an indicator of an individual having an allergy to aeroallergens such as house dust mite or pollen. We have edited the rationale for exclusion in Table 1 as follows: “ *More missing data and broader definition than allergy to food/drink*.”

- **Maternal smoking**: I would welcome arguments why information on maternal smoking was included in the final model. If I understand, it was only weakly related, and negatively (i.e. protective). Should I, as a GP, really take this into account and tend not to treat a child with ICS if the mum smokes? could the findings be explained by reverse causation (mothers stopped smoking when children became symptomatic) or by the chosen reference standard (doctors might have tended to prescribe antibiotics rather than ICS to children of smoking mums?).  At least this should be explained.

**Response: **For clarity, the variable included in the model was smoke exposure during childhood (rather than maternal smoking). The contribution of the variable in the model was unexpected and does not fit with a meta-analysis which investigated this topic ^^[iii]^^ so we do not feel it should be considered to reflect a ‘protective’ effect of smoking on asthma. One explanation might be that passive inhalation of smoke may have an inflammatory effect on the airway, which can increase the likelihood of lower respiratory tract infection[iv] and the propensity to wheeze.[v]Therefore in the prediction model, exposure to cigarette smoke may have reduced the probability of asthma because an alternative reason for symptoms was more likely. From a GP perspective, we don’t think it should influence GPs prescribing decisions, however, it might be that if a child / young person is presenting with lower probability of asthma, that clinicians seek further evidence by organising objective tests, rather than making a diagnosis based on history alone. The suggestion of reverse causation is also plausible. We have added the following to the discussion: *“In our study, childhood exposure to cigarette smoke was associated with a reduced probability of asthma which was unexpected given that a meta-analysis found household exposure to tobacco smoke was associated with an increased incidence of asthma in children. Passive inhalation of smoke may have an inflammatory effect on the airway which can increase the likelihood of lower respiratory tract infection and the propensity to wheeze. Therefore, in our model, exposure to cigarette smoke may have reduced the probability of asthma because an alternative reason for symptoms was more likely. Another explanation might be reverse causation, as it is possible that parents stopped smoking when their child developed asthma like symptoms.”*

- It is a pity that the model does only use information on previous symptoms (any time in life – yes/no) and previous lung function tests and prescriptions of asthma inhalers, but not more detailed information on the current health status (when the child presents to the GP to get the DX/ICS prescription, and the 12 months before. This could be more informative and improve the model further. Such clinical details (which could easily be assessed by the physician would be frequency of symptoms at day- and nigh-time, trigger factors, etc). I understand this is an inherent limitation of the dataset, but it could be further discussed in particular with relating to future research.

**Response: **We agree and have added the following to the discussion: *“The inclusion of predictors capturing information at the time of presentation (e.g., frequency and variation of symptoms or triggers) should be considered in future prospective research.”*

- **Final number of variables**: It seems that all variables that remained significant in a backwards selection were included in the model. I wonder why not a more robust variable selection (some shrinkage procedure, such as by the LASSO method) has been chosen, or why coefficients were not rounded. To my understanding, all this would have further reduced overfitting and made the model more robust and simpler. For instance, I am not sure if variables with very small coefficients (i.e. Table 3: smoking, social class, hayfever, eczema, allergy to food or drinks) substantially improve the model fit (sufficient improvement to justify making it more complicated).

**Response: **Yes, LASSO could have been used and was also suggested by a peer reviewer of the study protocol (https://wellcomeopenresearch.org/articles/5-50). As per our response then, we agree that LASSO could offer potential statistical advantages, but we chose to continue with our originally stated method, because we felt the clinical insight we brought to the study and the views of patient and public involvement members helped to limit the number of candidate predictors prior to modelling and reduce the chance of overfitting. In terms of variables with small coefficients, we opted to follow the methods as laid out in the protocol, and therefore chose to keep predictors that were selected during backward selection. The re-fitted model excluded social class and maternal asthma, and there was only a small difference to the AIC (full model = 5085.93, refitted model = 5094.33), however overall, we felt that the full model was parsimonious and uncomplicated. Additionally, we intended to implement the prediction model as a clinical decision support system which would help users to input information irrespective of the model complexity.

- **Describing model performance**: for the clinician, it would be helpful if additional measures were shown, such as sensitivity and specificity, NPV and PPV, for different scores.

**Response: **There are several metrics to describe model performance, and we chose to prioritise c-statistic, calibration slope, calibration plot based on the prediction modelling training received from experts from the TRIPOD group.[vi] Calculating other measures of performance such as sensitivity, specificity, negative and positive predictive values would have been another approach but would require a threshold / risk groups to be defined which we chose not to do.

**4. External validation: **

- The dataset used for external validation is very different from the one used for derivation, in that only information from GP records is available, while in the derivation sample they used also information form research questionnaires.  (see discrepancies in prevalence of predictors, Table 2). I think it would have been easier (and closer to the setting the model will be used in) if also the derivation dataset used only information form the health care records.

**Response: **When exploring opportunities for prediction model development in children, ALSPAC had advantages over other datasets because it had a number of variables that we hoped to include in a prediction model, for instance: FeNO, spirometry, skin prick testing, detailed reporting on symptoms. It was only during the data preparation phase that we realised many of the desired variables had been collected in a subset of the total cohort and were thus unavailable for use in the modelling due to missingness (as indicated in Table 1). Deriving the model using information only in health care records would have held advantages and disadvantages. Health care records rely on relevant data being coded. Unfortunately, information relevant for diagnosing asthma (such as symptoms, hay fever) are typically not well coded but more commonly held in free text data which are rarely available for research. Therefore, a prediction model derived using data only from health care records would lack detailed information on these predictors. Consequently, our approach was to first derive the prediction model in a dataset where information had been well recorded (i.e. ALSPAC in combination with data from health records), and then test the model in a dataset created from health records to see how well it performed in data closer to the setting in which the model will be used. As we mention in the discussion, one avenue we are pursuing is the use of unstructured data from health care records (i.e., free text) which could enhance the information available. We have added the following text to the discussion: *“We sought to externally validate the model in routinely collected data, because we hoped to learn how the model would perform in a dataset which closely represented routine primary care. However, the external validation dataset had substantial differences in the way that predictors were constructed and participant characteristics, which made it harder to directly compare model performance to the derivation dataset.”*

- I am not sure if the process described is really an external validation. (Steyerberg EW. Clinical Prediction Models : A Practical Approach to Development Validation and Updating. New York: Springer; 2009. doi:10.1007/978-0-387-77244-8). Rather, it seems the model was re-fitted in the new dataset.

**Response: **It was not possible to externally validate the full model (original model made in the derivation dataset) because we were unable to obtain equivalent variables for social class and maternal asthma in the external validation dataset. Subsequently, a second model excluding social class and maternal asthma was fitted (we termed this the “re-fitted” model) in the derivation dataset. Therefore, whilst not exactly as we had initially planned, we were able to complete an external validation, but it was of the re-fitted model rather than the full model. 

- In my (statistically limited) understanding it would have been more logical to use in the derivation cohort only those predictors which are available in both cohorts, fit the model only with these, and see how the original model performs in the validation cohort .

**Response: **Ultimately using only the predictors available in both cohorts was what we did with the re-fitted model. We might have chosen to report only the re-fitted model but we felt it important to report the steps that we took and had outlined in the protocol. **Minor:**

- For the reader, it would be good to shortly describe/explain  the asthma Read code in the paper

**Response: **The code list for the outcome measure is available in the extended data (as per the journal requirements). The link is here: https://osf.io/kfz3n/ It is included in the manuscript as reference 15.

- Could you tell the reader how many doses are contained in an average UK ICS prescription; i.e. for how many months of twice-daily treatment will the three prescriptions last?

**Response: **This varies quite a lot depending on inhaler type, doses and adherence. However, in general most inhaler types would be anticipated to last one month if taken every day. We’ve added the following text to the methods: *“Participants who received at least three prescriptions of an ICS (which, if used every day would typically last one month), as a single inhaler or combined with a long-acting beta agonist, on separate days within a one-year period were identified.”*

- Table 4: please specify if the data for this table come from the derivation or validation cohort.

**Response: **It is from the derivation cohort. We have updated the heading for Table 4.

- Please describe better at which time-points the predictor data were selected, and (in ALSPAC), how much information was provided by questionnaires and by health care data.

**Response: **We have added more detail in Table 1. 

 Saying all that, the paper has been well done and transparently describe and very much deserves publication after some revision. 

 ​​​​​​​PS. I base my comments mainly on the suggestions by Steyerberg, citation below (or what I understood from it)  

**Response: **Thank you, we appreciate your comments.  

**References** [i] Health Improvement Scotland: BTS/SIGN British Guideline for the management of asthma. SIGN 158,2019 [ii] Daines L, McLean S, Buelo A, et al.: Systematic review of clinical prediction models to support the diagnosis of asthma in primary care. *NPJ Prim Care Respir Med.* 2019;29(1):19. 31073125 10.1038/s41533-019-0132-z 6509212 [iii] Burke H, Leonardi-Bee J, Hashim A, Pine-Abata H, Chen Y, Cook DG, Britton JR, McKeever TM. Prenatal and passive smoke exposure and incidence of asthma and wheeze: systematic review and meta-analysis. *Pediatrics*. 2012; 129(4):735-44. [iv] Li JS, Peat JK, Xuan W, Berry G. Meta‐analysis on the association between environmental tobacco smoke (ETS) exposure and the prevalence of lower respiratory tract infection in early childhood. *Pediatric pulmonology*. 1999;27(1):5-13. [v] Lux AL, Henderson AJ, Pocock SJ. Wheeze associated with prenatal tobacco smoke exposure: a prospective, longitudinal study. *Archives of disease in childhood*. 2000;83(4):307-12. [vi] Collins GS, Reitsma JB, Altman DG, et al.: Transparent Reporting of a multivariable prediction model for Individual Prognosis Or Diagnosis (TRIPOD). *Annals of Internal Medicine.* 2015;162(10):735–6.
